# Efficacy of anchoring the four-arm transvaginal mesh to the mid-urethra vs original surgery as a surgical correction for stress urine incontinence in coexisting anterior vaginal prolapse grades II and III: study protocol for a randomized controlled trial

**DOI:** 10.1186/s13063-017-2314-8

**Published:** 2017-12-28

**Authors:** Zoltán Fekete, Andrea Surányi, Lórand Rénes, Gábor Németh, Zoltan Kozinszky

**Affiliations:** 1 Division of Urogynaecology, Department of Obstetrics and Gynaecology, University of Szeged, Semmelweis u. 1., 6725 Szeged, Hungary; 2 Department of Obstetrics and Gynaecology, Blekinge Hospital, Karlskrona, Sweden

**Keywords:** Modified transvaginal mesh, Complications, SUI with POP–Q II–III, IUGA classification

## Abstract

**Background:**

The prevalence of obesity with aging is escalating alarmingly; and pelvic organ prolapse (POP) and stress urinary incontinence (SUI) are now becoming a growing epidemic among the elderly. Synthetic transvaginal mesh has been employed with increasing popularity in the treatment of POP and is usually highly effective in controlling the principal symptoms of prolapse. However, studies have reported that mesh operations provide fairly unfavorable SUI cure rates. Therefore, additional anti-incontinence surgical strategies are increasingly being scrutinized to achieve better postoperative continence without any significant side-effects for patients with both POP and SUI. We hypothesize that the modification with the fixing of the mesh to the mid-urethra is superior to the original transvaginal mesh operation (TVM) with regard to anti-incontinence.

**Methods:**

One hundred and thirty patients diagnosed with POP–Q II–III and concomitant SUI requiring surgical treatment will be included in this prospective, randomized, double-blind, controlled clinical trial. Patients will be randomly allocated to receive either original TVM (TVM group, *n* = 65) or modified TVM surgery (mTVM group, *n* = 65). As the primary outcome parameter, we will evaluate the objective SUI and POP cure rates. Secondary endpoints include postoperative morbidity as assessed with the International Urogynaecological Association classification and subjective prolapse and incontinence cure rates reported by questionnaires.

**Discussion:**

Recognizing the importance of an additional surgical procedure for anti-incontinence management, we aim to investigate whether a stabilizing suturing of the mesh to the mid-urethra delivers superior SUI correction compared to the original prosthesis surgery.

**Trial registration:**

ClinicalTrials.gov, NCT02935803. Registered on 20 May 2016.

**Electronic supplementary material:**

The online version of this article (doi:10.1186/s13063-017-2314-8) contains supplementary material, which is available to authorized users.

## Background

Demand for pelvic organ prolapse (POP) and stress urinary incontinence (SUI) surgery is expected to increase due to an expanding rate of obesity among elderly women. POP is defined as the protrusion of the pelvic organs from the normal anatomical location toward or through the vaginal opening; the current prevalence of symptomatic POP is in the range of 3–8% [1, 2]. SUI, classified as involuntary loss of urine during physical activities and a resultant increase in intra-abdominal pressure, is also highly prevalent, reaching as high as 24.8% [3].

Also of note, one-fifth of women in the United States receives surgery either for SUI or for POP [4], where the cumulative risk for SUI surgery is 13.6% and that for POP surgery is 12.6% [4]. Both pathologic conditions develop in > 50% of the women affected [5]. Synthetic transvaginal mesh (TVM) has been increasingly employed in the treatment of POP and tension-free slings are useful in the management of incontinence. Synthetic mesh during repair has principally been used due to higher efficacy compared to that of native tissue repair and resorbable mesh; however, complications appear to be more prevalent [6–9]. Common complications include mesh extrusion, chronic pelvic pain, dyspareunia, and infection [7–10]. The anti-SUI efficacy of the prosthetic placement is barely 72–83% [11–13]; however, it is assumed that a combination of a synthetic mesh with the sling operation [5, 14–16] will substantially increase the cure rate for concomitant SUI.

Despite the increased consideration of the combined surgery for both genitourinary pathologies in one session, there is a lack of consensus on the optimal treatment. It is supposed that a combined operation with mesh and sling is highly effective for the treatment of POP and SUI; however, the complication rate is elevated [5, 14–16].

Therefore, the research group developed a modification to the transobturator four-arm TVM [13, 17] to increase its anti-incontinence effect. While the sling is located beneath the mid-urethra, the TVM elevates the distal part of the anterior vaginal wall [13, 17]. In the original TVM, the posterior part of the mesh is anchored to the anterior aspect of the cervix and the anterior arms are spread under the bladder neck with stabilizing sutures. We hypothesize that the original TVM operation can be followed by residual SUI since the strengthening of the back arms may result in a backward dislocation of the entire mesh. The posterior movement of the mesh allows the dorsal rotation of the urethra since the mid-urethra is not suspended. The proposed modification to the original surgical procedure includes the suture of the anterior part of the mesh to the mid-urethra to prevent the mesh sliding. We think that the appropriate elevation of the mid-urethra would thus occur with the anterior arms and that would achieve a more effective anti-incontinence. The pubourethral ligament is usually loose in SUI, but the anchored mesh would theoretically normalize its function and stabilize the urethra. Intra- and postoperative complication rates would be expected to be similar to those with the original four-arm TVM, but the modified TVM with the anchoring suture would be slightly superior with respect to POP repair and remarkably more effective in SUI correction. In our preliminary study, the anterior fixing of the TVM to the mid*-*urethra demonstrates as high an efficacy of anti-incontinence as 96.8% and an enormously reduced recurrence prolapse rate of 3.2% in (unpublished data). The mesh extrusion rate is particularly low and this may be due to the fact that the stabilizing sutures exert a lack of “folding/wrinkling” of the edge of the mesh, preventing a lifting up of the mesh which does not compress the mucosa and derange the periprosthetic vasculature.

A further modification to the TVM surgery is that the positioning of the mesh will occur 1.5 cm below the urethral meatus, leading to an elevation of the entire anterior vaginal wall including the anterior and middle compartments as well. By contrast, the original TVM surgery does not prevent anterior compartment prolapse [13, 17].

## Methods/design

### Study design

**Fig. 1:**
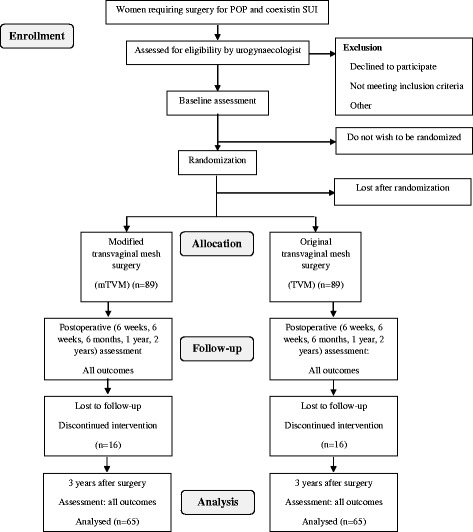
CONSORT study flow *diagram*

The present study is a single-center, prospective, double-blind (participant, investigator/surgeon, outcome assessor), randomized, controlled trial. The study will be conducted in accordance with the Declaration of Helsinki and has been approved by the local medical ethics committee at the University of Szeged under reference number 55/2016. The trial is registered under NCT 02935803, and patient recruitment started on 22 August 2016. The trial flow diagram is presented in Fig. 1 (CONSORT study flow diagram). The protocol follows the Standard Protocol Items: Recommendations for Interventional Trials (SPIRIT) checklist (see Additional file 1).

#### Patient recruitment and consent procedure

Patients will be recruited from the urogynecology consultation at the Division of Urogynaecology, Department of Obstetrics and Gynaecology, University of Szeged, Hungary. All study participants will be provided an information sheet and a consent form describing the study in brief so they can decide whether to participate in the study. Each participant will be explicitly informed that participation in the study is voluntary, that she may withdraw from the study at any time and that withdrawal of consent will not affect her subsequent medical assistance and treatment.

##### Consent to publish

Written informed consent was obtained from the patients for publication of their individual details and accompanying images in this manuscript. The consent form is held by the authors in the patients’ clinical notes and is available for review by the Editor-in-Chief.

#### Participants considered for trial

This will be a prospective longitudinal study involving all patients successively scheduled for operation for symptomatic prolapse POP–Q grade II or III and coexisting SUI, who will be included in the study after informed consent has been obtained. Patients will be recruited in the Department of Obstetrics and Gynaecology at the University of Szeged. The study will be conducted for an estimated maximum of 18 months (between August 2016 and December 2017). Table 1 provides an overview of the patient recruitment and evaluation plan.

**Table 1:** Schedule of assessments/data collection

Assessment	Recruitment before intervention phase	Intervention (surgery)	Follow-up				
			6 weeks	6 months	1 years	2 years	3 years
Assessment of eligibility criteria	x						
Written informed consent	x						
Gynecological examination: incontinence symptoms	x	x	x	x	x	x	x
Gynecological examination: prolapse	x	x	x	x	x	x	x
Urodynamic examination	x				x		x
Adverse events		x	x	x	x	x	x
Questionnaires: PISQ-12 and PFDI	x		x	x	x	x	x
Introital sonography	x				x		x
Urine culture	x			x	x	x	x

The symptomatic POP–Q Stage II–III (determined by the gynecological examination using the International Continence Society quantification system) [18] anterior vaginal wall prolapse is defined as the maximum extent of the prolapsed anterior and middle compartments being within 1 cm above and 6 cm below the hymen [19, 20]. According to the international POP guidelines (the EBU and NICE guidelines) [21, 22], if the condition disrupts the patient’s life and non-surgical treatment options have not helped, it should be treated surgically.

In all cases, SUI will be visualized after a complete physical examination is performed (verified by pad test/Bonney test/two-dimensional [2D] introital sonography and urodynamic examination). The severity of SUI was assessed using the Ingelman–Sundberg classification [23]. Urodynamic examinations comprising uroflowmetry, cystometrography, the pressure-flow study, and the abdominal leak point pressure test will be performed before surgery to objectively determine the coexisting symptomatic SUI based on the international guidelines (the EBU and NICE guidelines) [21, 22]. The abdominal leak point pressure test will be used as a standardized examination method for the evaluation of SUI with urine leakage as a sign. If the intra-abdominal pressure recorded at the point of urine leakage was < 40 cmH_2_O, the origin of the SUI was set as intrinsic sphincter deficiency (ISD) [24]. In the case of ISD, preoperative pelvic floor training (PFMT) will be recommended. If the patient is unwilling to participate in PFMT or if the training was unsuccessful, we will recommend mesh surgery. This will also be the case for suspected urethral hypermobility, i.e. if the intra-abdominal pressure at the point of urine leakage was > 60 cmH2O.

#### Introital ultrasound findings

In all cases, introital ultrasound (GE Voluson 730) will be performed with a standardized bladder-filling volume of 300 mL. The vaginal probe (5–9 MHz) will be placed in the area of the vaginal introitus at the level of the external urethral orifice, with the patient in a semi-sitting position. Ultrasound assessment of the bladder and urethra starts in the mid-sagittal plane.

During the sonography, we measure the longitudinal (L) distance between the bladder neck and the line through the lower edge of the pubic symphysis and the horizontal (H) distance between the bladder neck and the upper edge of the symphysis. The two distances are measured at rest (L1, H1), during contraction (L2, H2), on pressing or while coughing (L3, H3). Changes in these parameters during contraction of the levator muscle and on pressing serve to evaluate the reactivity of the pelvic floor muscles and the adequacy of the supportive structures of the urogenital organs [25]. The funneling of the proximal urethra during coughing as a typical stress urinary sign also will be examined [25].

### Inclusion and exclusion criteria

#### Inclusion criteria

Female adults aged > 40 years with coexisting pelvic floor defects will be recruited, at least one year following delivery, irrespective of parity and pre- or postmenopausal state, medically and physically fit for the measurement and therapeutic surgeries, and, in the case of systemic or local estrogen treatment, stable for the past three months before inclusion.

#### Exclusion criteria

Exclusion criteria are: urge, mixed incontinence or occult SUI; prolapse < grade II or > grade III POP–Q ([21, 22], https://uroweb.org/guideline/urinary-incontinence), apical or posterior compartment prolapse, dysuria (bladder tumor, neurogenic urinary bladder damage), a history of mesh use or anti-incontinence pelvic procedures; pregnancy (urine test to accomplish); lactation period not yet finished; current urinary tract or vaginal infection, menstruation on the day of examination; contraindications for measurements or interventions, for example, acute inflammatory or infectious disease, tumor or fracture; de novo systemic or local estrogen treatment (< 3 months); de novo drug treatment with anticholinergics or other bladder active substances (tricyclic antidepressants and selective serotonin reuptake inhibitors) and cancer of the pelvic organs.

#### Concealment of group allocation from participants

After the screening phase, patients will be randomly assigned to one of the two therapy groups (the TVM group or modified TVM (mTVM) group). The allocation sequence will be generated by the independent urogynecology secretariat using online randomization software (http://randomization.com); allocation ratio = 1:1 (TVM group:mTVM group). The allocation will be concealed in sealed, opaque, sequentially numbered envelopes, which will be stored at the operating theatre. All the women recruited will be numbered consecutively corresponding to the numbered envelopes. The envelope will not be opened until the general narcosis of the study participant has been established. To increase the validity of the trial, the assigned procedure will be blinded for the study participants throughout the follow-up period. The participants will be informed of allocated treatment after completing the study forms 12 months after the procedures. If major complications occur, the study participants and the outcome assessor will be also informed of the allocated treatment at the time of any suspected complication(s).

### Randomization

The patients will be randomized to one of the study groups using a computer-generated list. Allocation concealment will be ensured by enclosing assignments in sealed, opaque, sequentially numbered envelopes, which will only be opened when the general narcosis of the study patient has been established [26].

Postoperative outcomes and sequalae will be assessed by an outcome assessor who is a gynecologist in a subspecialization program in urogynecology and also well trained in the transvaginal mesh operation. The outcome assessor will remain blinded to the type of intervention throughout the study.

#### Blinding

Participants will be blinded against the type of TVM surgery received (original TVM vs mTVM). The participant information document will not provide any information on the differences in surgical protocols such that the women could ascertain their group allocation. All investigators involved in data acquisition, data analyses, and statistics will also be blinded against group allocation. The surgeons in charge of the therapy cannot be blinded against group allocation and therefore will not be involved in data acquisition, data reduction, data analyses, or statistics.

### Measurement outcomes

Baseline (before intervention phase) and follow-up measurements (of primary, secondary, and tertiary outcomes) after six weeks to three years will be performed at the Division of Urogynaecology, Department of Obstetrics and Gynaecology, University of Szeged, Hungary, by an experienced urogynecologist who will be blinded to group allocation of participants and who will not operate on the patients (Table 1).

### Primary endpoints

The primary outcome measures will be a significant improvement in POP repair and objective cure of SUI after the surgery. The efficacy of POP repair will be understood as a significant (> 3 cm) improvement during follow-up at points Aa, Ba, C, and D using the POP–Q system (International Continence Society) [19, 20]. Anti-incontinence efficacy is classified as no further SUI, as diagnosed by cough tests and urodynamic examinations. Besides the gynecological and urodynamic examinations, sonographic findings from introital ultrasound examinations will be analyzed in terms of anatomical success both before and following surgery and during follow-up.

### Secondary and tertiary endpoints

The secondary measurement outcome will comprise the intraoperative findings and postoperative factors. As concerns the long-term postoperative complications of the mesh procedures, we will determine the extrusion rate, the presence of de novo urge symptoms (DNUS) or urinary tract infection (UTI), and the need for reoperation. The diagnosis of DNUS will be set if detrusor pressure changes are detected in cystometrographic pressures after the surgeries. The postoperative complications that will lead to reoperation will be infection, recurrent descent or incontinence, implant extrusion, chronic pelvic pain, and total retention. Operative and perioperative complications (six weeks after the procedures) described after TVM vs mTVM will be collected; overall frequency within all the cases will be calculated and severity will be graded using the IUGA classification comprising all the follow-up periods [27].

The subjective cure for prolapse and incontinence will be measured with a significant enhancement of the Pelvic Organ Prolapse/Urinary Incontinence Sexual Questionnaire (PISQ-12) and Pelvic Floor Distress Inventory (PFDI) scores. The PISQ-12 and PFDI are validated to assess the impact of SUI symptoms on quality of life and sexuality and relate well to the prolapse symptoms. Previous research has demonstrated that the questionnaires correspond well with grade of prolapse and urodynamic findings [5, 13, 17]. Our research group has assessed the validity of the questionnaires in screening for subjective genitourinary symptoms (unpublished data). To guarantee blinding of the tertiary outcome, the participant will complete the questionnaire without the outcome assessor present and seal it in an envelope, which will be given to research staff. The subjective outcome assessment is largely performed by participant-completed questionnaire, thus avoiding interviewer bias.

### Trial interventions

Participants will receive the allocated intervention, either the original TVM operation or mTVM surgery. The surgical interventions will be delivered by two surgeons with expertise in the specific intervention and subspecialized in urogynecology. They will not assess the measurement outcomes. Further details on the interventions are provided below.

### Original transobturator four-arm transvaginal mesh

Original transvaginal subvesical mesh operations will be performed as described earlier by Sergent et al. [13, 17]. The operative technique is described in detail in another study [28]. The routine surgical technique will consist of a longitudinal incision of the anterior vaginal wall throughout its thickness from 3 cm below the urethral meatus to the cervix. The posterior part of the mesh will be anchored to the anterior side of the cervix using two non-absorbable Prolene® 2-0 sutures (Ethicon, Issy-les-Moulineaux, France), while the mesh will then be spread by securing its anterior parts beneath the bladder neck using two or three Monocryl® 2-0 absorbable sutures (Ethicon, Issy-les-Moulineaux, France). Conventional instruments will be employed for the original TVM procedure.

### Modified intervention surgery

In the modified surgical technique, the prosthesis is placed between 1.5 cm below the urethral meatus and the cervix and the anterior part of the mesh is anchored with a stabilizing suture to the periurethral tissue at the level of the mid-urethra to elevate the middle part of the urethra, leading to potentially more effective anti-incontinence. An additional document file presents the intervention (detailed description of the mTVM) in detail (see Additional file 2).

### Assessment of safety: postoperative complications/reoperations

In the current study, there are no anticipated risks or inconveniences, as the examinations and intervention employed are well-known and widely used in pelvic floor defect surgery. The modification to the TVM surgery does not carry a higher risk for patients than that of the original TVM in the setting of a fully equipped operation theatre. This makes the immediate detection and treatment of adverse events possible. Also, after leaving the operation room, all patients will be closely monitored for the occurrence of potential (severe) adverse events (short-term postoperative complications) on the postoperative intensive ward. Moreover, the inclusion of each individual patient in the study is indicated in the electronic hospital information system and, hence, is visible to all physicians and nurses involved in the care of the patient. This facilitates the reporting of (severe) adverse events to the principal investigator. The principal investigator will report suspected unexpected serious adverse reactions to the Institutional Review Board.

## Statistical methods

### Hypothesis

Alternative hypothesis for primary outcome: it is hypothesized that the group undergoing the modified transvaginal mesh operation will have a statistically higher improvement of continence measured by gynecological and urodynamic examination, and from the questionnaire administered before and after the intervention phase.

#### Sample size calculation

As we have newly developed the modification to the prosthesis surgery, an exploratory pilot study was designed to evaluate the feasibility of the modification to the mesh for the treatment of SUI. Twenty patients with SUI and POP were recruited for a mTVM operation by the same two senior surgeons who are conducting this randomization study. The sample size calculation study was designed based on preliminary data on the 20 patients. The newly developed technique yielded an objective SUI cure rate of 92% as opposed to 72% for the original TVM published by Sergent et al. [13, 17]. Sample size calculations were performed with G*Power software [29], using the statistical model for an χ^2^ approach. Consequently, sample size was estimated theoretically and an effect size of = 0.1, indicating a small effect, will be accepted. The sample size was calculated for the primary outcome of the SUI cure rate with the following assumptions: α = 0.05, power (1–β error probability) = 0.8, number of groups = 2. Based on these assumptions, a total sample size of *N* = 130 was estimated. In anticipation of dropouts (10%: *n* = 16) or a violation of protocol (10%: *n* = 16), a final sample size of *N* = 162 (81 participants per group) results.

### Statistical analyses

Analysis of the patients will follow the CONSORT flow diagram (Fig. 1) through the phases of the study (enrollment [assessed, excluded, randomized], allocation [control group and experimental group with intervention received or not received], follow-up [lost to follow-up, discontinued intervention] and analysis) [30].

All statistical analyses will be conducted using SPSS software version 22 (IBM, Armonk, NY, USA). All tests will be two-sided and significance will be set at *P* < 0.05. Efficacy measurements were adjusted by intention-to-treat analysis. Missing values will be replaced using the last observation carried forward (LOCF) method. No subgroup analyses are planned. Standard deviations, 95% confidence intervals, and median will be used for the descriptive analyses. Primary and secondary outcome analysis: the Chi-square test or Fisher’s exact test will be employed to identify any objective outcome differences among groups.

Generally, continuous data will be checked for normality using the Shapiro–Wilk test. If the normality assumption is violated, then PISQ-12 and PFDI scores as tertiary outcomes will be normalized by log transformation (log10(x)). Univariate-repeated measure analysis of variance (ANOVA) will be used to determine the secondary outcome between and within the two groups (mTVM, TVM group) at six endpoints (before intervention and during follow-up clinical appointments 1–5 following intervention). Mixed design ANOVA will be carried out to determine the effects of the modified operation on subjective cure rate for POP and SUI, and the Bonferroni post hoc test will be used to test the difference between means. All statistical analyses will be completed after the final measurement of the last patient during the last clinical appointment after intervention. The repeated measure design with seven points in time allows us to monitor how patients change over time in both short-term (before/during intervention) and long-term situations (before/after intervention).

## Discussion

Coexisting POP and SUI are increasingly recognized as a major health and financial concern affecting 63–80% of postmenopausal women [31]. TVM is the standard surgical method for the anatomical restoration of middle compartment Stage II–III prolapse; however, it should be supplemented with a mid-urethral sling to achieve better SUI treatment. Moreover, the combined mesh and sling operations yield unfavorably more frequent complication rates and may provoke voiding dysfunction and recurrent UTI. Furthermore, following the original TVM, some residual SUI can develop because of the backward dislocation of the mesh.

To the best of our knowledge, the present study is the first to investigate a surgical modification to TVM for more effective anti-incontinence. Should this newly developed modification be proved successful in treating SUI, it could be introduced in clinical practice due to its simplicity.

## Additional files

Additional file 1: SPIRIT 2013 checklist: recommended items to address in a clinical trial protocol and related documents. (DOC 127 kb)

Additional file 2: Intervention surgery. (DOC 30 kb)

## Abbreviations

ANOVA: Analysis of variance
LOCF: Last observation carried forward
mTVM: Modified transvaginal mesh operation
PFDI: Pelvic Floor Distress Inventory
PFMT: Pelvic floor training
PISQ-12: Pelvic Organ Prolapse/Urinary Incontinence Sexual Questionnaire
POP: Pelvic organ prolapse
POP-Q: Pelvic organ prolapse quantification system
SUI: Stress urinary incontinence
TVM: Transvaginal mesh operation
UTI: Urinary tract infection
