# Supplementary material for: Efficacy of anchoring the four-arm transvaginal mesh to the mid-urethra vs original surgery as a surgical correction for stress urine incontinence in coexisting anterior vaginal prolapse grades II and III: study protocol for a randomized controlled trial
Source: Trials. 2017 Dec 28;18:624. doi: 10.1186/s13063-017-2314-8 (PMC5746023; doi:10.1186/s13063-017-2314-8)
Supplement: Supplementary file 2 — Intervention surgery. (DOC 30 kb) [file 13063_2017_2314_MOESM2_ESM.doc]

**Additional file 2**

**Intervention surgery**

All operations will be carried out with 100% polypropylene monofilament meshes produced by Aspide® SURGIMESH® PROLAPSE (Aspide Médical, La Talaudière, France). The implanted vaginal prosthesis has pores which are 1.6 x 1.7 mm in size and is approved for anterior vaginal repair.

Surgery will be performed under general anesthesia with the patient in the lithotomy position and with an indwelling 16Ch urinary catheter. The anterior vaginal wall will incised longitudinally throughout its thickness from the 1.5 cm below the urethral meatus (where the mid*-urethra* located*)* to the cervix. The thickness of the dissection, the location of the vaginal incision, the placement of the mesh and the closure of the incision will vary only minimally, and the length of the incision intended to be be 6-7 cm. All the operations will be performed by two experienced senior surgeons who are subspecialists in urogynaecology. Our pilot study revealed that the differences in the operative characteristics including the length of the incision and the thickness of dissection varies only minimally between the two surgeons (Cohen’s kappa: 0.89 and 0.97, respectively).

Before insertion, all sterile meshes will be soaked in iodic fluid (Betadine®). The Surgimesh® device will be introduced beneath the dissection and the four arms of the mesh will be then passed through the obturator membrane. The posterior part of the mesh will be anchored to the anterior side of the cervix using two Prolene® 2-0 sutures (Ethicon, Issy-les-Moulineaux, France).

The mesh will be spread then by securing its anterior parts beneath the mid*-urethra* using two Vicryl 2-0® absorbable sutures (Ethicon, Issy-les-Moulineaux, France). We hypnotize that this manouver will promote the proper elevation and closure of the *urethra*. The mesh will be adjusted then in a tension-free manner beneath the distal part of the urethra and bladder. Lastly, the anterior vaginal will beclosed using Monocryl® 3-0 absorbable sutures (Ethicon, Issy-les-Moulineaux, France), with slight colpectomy. Prophylactic preoperative antibiotics (cefazolin 1g, amoxicillin and clavulanic acid 1.2g or gentamycin 160mg) will be administered intravenously. A urinary catheter will be removed on the morning of the postoperative day. A vaginal gauze pack (gauze soaked in Betadine iodine) will be placed for 12h. The post-voided residual urine will measured by ultrasonography before each patient was discharged. All the patients will obtain topical intravaginal oestrogen cream treatment for at least twelve months following the operation (Ovestin 1mg/gram daily).
